# Layer by layer preparation of Fe_3_O_4_@Cg-DTC/AgNPs as colloidal antimicrobial and anti-biofilm agent

**DOI:** 10.1038/s41598-025-29960-w

**Published:** 2025-12-02

**Authors:** Solmaz Ohadian Moghadam, Lida Lotfollahi Hagghi, Reza Taghavi, Mohammad Reza Nowroozi, Ziba Karimi, Amir Hasanzadeh, Sadegh Rostamnia

**Affiliations:** 1 Uro-Oncology Research Center, Tehran University of Medical Sciences, Tehran, 14197-33141 Iran; 2 Department of Microbiology and Virology, School of Medicine, Urmia University of Medical Sciences, Urmia, 57157-99313 Iran; 3 Advanced Porous Materials and Energy Technology (APMET) Res. Lab., Department of Chemistry, Iran University of Science and Technology (IUST), Tehran, PO Box 16846-13114 Iran

**Keywords:** Magnetic NPs, Ag NPs, Dithiocarbonate functionalization, Antibacterial, Anti-biofilm, Biotechnology, Materials science, Microbiology, Nanoscience and technology

## Abstract

To solve antimicrobial resistance in bacteria, metal nanoparticles (NPs) have gained tremendous attention due to their effectiveness and ease of use. Here, we prepared a core–shell dithiocarbonate carbon functionalized magnetic composite and employed it to support and stabilize the silver nanoparticle (Ag NPs). A chemical reduction method was employed to decorate the Ag NPs over the surface of the magnetic composite. The prepared materials were characterized using FT-IR, XRD, VSM, EDX, SEM, TEM, particle size distribution analysis, and elemental mapping. The results demonstrated that the designed support effectively prevented aggregation of the Ag NPs, ensuring their uniform dispersion throughout the structure. The Ag NPs decorated core–shell material was used as an antimicrobial agent against *Escherichia coli*, *Pseudomonas aeruginosa*, and *Staphylococcus aureus.* The minimum inhibitory concentrations of nanocomposite against *Escherichia coli*, *Pseudomonas aeruginosa*, and *Staphylococcus aureus* were 0.58 ± 0.38, 1 ± 0, and 4.67 ± 3.06 µg mL^−1^, respectively. The results demonstrated that Fe_3_O_4_@C_g_-DTC/AgNPs nanocomposite has a stronger antibacterial effect than the control antibiotic, ciprofloxacin. Regarding biofilm inhibition, the lowest concentration of Fe_3_O_4_@Cg-DTC/Ag composite required to inhibit biofilm formation in *E. coli* was 0.0625 µg mL^−1^, while the concentrations required for *P. aeruginosa* and *S. aureus* were 0.5 and 1 µg mL^−1^, respectively. Cytotoxicity assays confirmed that the nanocomposite did not induce toxicity in NIH-3T3 cells. Overall, these results demonstrate the potent antibacterial and anti-biofilm activity of the Fe_3_O_4_@Cg-DTC/AgNPs nanocomposite while maintaining cytocompatibility.

**Supplementary Information:**

The online version contains supplementary material available at 10.1038/s41598-025-29960-w.

## Introduction

Antibiotic resistance in bacteria is a growing global concern, posing a significant threat to public health^1–7^. The overuse and misuse of antibiotics have led to the development of resistant strains of bacteria that no longer respond to traditional antibiotic treatments^8^. This has serious implications for the effectiveness of antibiotics in fighting infections and diseases, potentially leading to higher mortality rates and increased healthcare costs^9^.

The emergence and spread of drug-resistant bacterial strains have become a critical concern in the management of infectious diseases globally^10^. These challenges have led to increased treatment failures, complications, and pose significant risks to public health^11^. Organisms involved in these infections include *Escherichia coli* (*E. coli*), *Pseudomonas aeruginosa* (*P. aeruginosa*), *Staphylococcus aureus* (*S. aureus*), etc^12^. To prevent such infections, antibiotic prophylaxis with fluoroquinolones is performed. Owing to the growing resistance to fluoroquinolones due to misuse and overuse, the need to seek appropriate alternatives is felt^13^.

One promising approach to address antibiotic resistance is the use of metal nanoparticles (NPs), which possess unique physicochemical properties that enable them to effectively combat resistant bacteria. Among them, silver nanoparticles (Ag NPs) have shown strong antimicrobial activity against a wide range of bacteria, including multidrug-resistant strains^14^. Their antibacterial properties arise from multiple mechanisms such as disruption of bacterial membranes, interference with cellular functions, induction of oxidative stress, and the release of silver ions ^14^. The nanoscale size of Ag NPs also provides a high surface area-to-volume ratio, enhancing their interaction with bacterial cells and further improving their efficacy ^15^. These properties have led to the incorporation of Ag NPs into wound dressings, medical devices, and household products.

Despite these advantages, the uncontrolled release of silver ions and the tendency of Ag NPs to aggregate during conventional synthesis limit their stability and may cause cytotoxic effects^18‚19^. To overcome these challenges, stabilizing strategies such as the use of polymers, carbon-based coatings, and core–shell nanostructures have been proposed. Core–shell architectures, in particular, offer low toxicity, high solubility, and thermal stability, making them attractive for biomedical applications. Magnetic nanoparticles (MNPs), especially iron oxide-based systems, are widely studied due to their role in antimicrobial activity, which is attributed to reactive oxygen species generation, membrane disruption, and ion release^22^. In addition to antibacterial properties, magnetic nanomaterials show promise for broader biomedical applications, including imaging, drug delivery, hyperthermia therapy, and even anticancer strategies^23^. MNPs also exhibit intrinsic antimicrobial properties through several mechanisms, including generating reactive oxygen species (ROS) that induce oxidative stress in bacterial cells, disrupting the integrity of bacterial membranes by direct interaction, and releasing metal ions that interfere with essential cellular processes.

Despite the availability of various nanoparticles, current strategies for addressing antibacterial resistance in bacteria still face major challenges^24^. The emergence of new drug-resistant bacterial strains, aggregation of nanoparticles, uncontrolled release of metal ions, and limited anti-biofilm activity are some of the shortcomings of current methods^25^. Furthermore, many nanoparticles lack multifunctionality, which has proved to be an effective approach to enhance targeted delivery in biomedical applications.

Various commercial metal oxide nanoparticles, including ZnO, TiO₂, Ag, Cu, as well as composite systems such as ZnO/TiO_2_, CuO/TiO_2_, and ZnO/CuO, have been widely investigated for their antibacterial properties^26‚27‚28,29,30^. While these materials exhibit significant antimicrobial activity, they often suffer from limitations such as aggregation, uncontrolled ion release, cytotoxicity, and limited anti-biofilm effects.

In this study, we developed a magnetic composite with a core–shell structure functionalized with dithiocarbonate. The designed magnetic composite was utilized to stabilize silver nanoparticles (Ag NPs). The resulting Ag NP-coated core–shell material functioned as an antimicrobial agent against Escherichia coli, Pseudomonas aeruginosa, and Staphylococcus aureus. The findings revealed that the Fe_3_O_4_@Cg-DTC/AgNPs nanocomposite exhibited a stronger antibacterial effect compared to the control antibiotic, ciprofloxacin. Furthermore, the anti-biofilm activity assessment indicated a significant anti-biofilm effect. A cytotoxicity assay further confirmed that the nanocomposite was safe for cell lines, highlighting its potential for therapeutic applications. The MTT assay demonstrated that Fe_3_O_4_@Cg-DTC/AgNPs exhibited low cytotoxicity toward NIH-3T3 cells, with only a 22% reduction in viability at the highest tested concentration (400 µg/mL), indicating good biocompatibility. The Fe_3_O_4_@Cg-DTC/AgNPs nanocomposite offers clear advantages over conventional silver nanoparticles by preventing aggregation, enabling controlled ion release, and exhibiting strong antibacterial and anti-biofilm activity with good biocompatibility. Its magnetic nature also opens possibilities for multifunctional biomedical applications. However, reduced magnetic saturation, partial loss of monodispersity, and challenges in large-scale production highlight the need for further optimization and long-term evaluation.

## Experimental

### Materials

All the applied materials in this manuscript were purchased from commercial sources and used without further purification. The cells (NIH/3T3) were purchased from the Pasteur Institute of Iran.

### Preparation of Fe_3_O_4_ magnetic nanoparticles

A hydrothermal method was employed for the preparation of MNPs. First, 1.5 g FeCl_3_·6H_2_O, 2 g sodium acetate, and 1 g polyvinyl pyrrolidone (PVP) were added to a 50 mL beaker and stirred for 2.5 h. Then, the obtained uniform solution was transferred to a Teflon-lined stainless autoclave and heated at 200 °C for 10 h. The black residue was separated from the solution by an external magnet and washed 3 times with water and ethanol. The obtained MNPs were dried at 60 °C overnight in an oven.

### Preparation of carbon-coated Fe_3_O_4_ magnetic nanoparticles

Preparation of the Fe_3_O_4_@C_g_ (glucose drives carbon) was carried out via a hydrothermal process. The obtained Fe_3_O_4_ MNPs were dispersed in 30 mL of deionized water containing 5 g of glucose under sonication for 30 min. Next, the obtained solution was transferred to a Teflon-lined stainless autoclave and heated at 200 °C for 12 h. The obtained core–shell magnetic Fe_3_O_4_@C_g_ was separated from the solution using an external magnet, washed with deionized water and methanol three times, and dried overnight in an oven.

### Dithiocarbamate functionalization of Fe_3_O_4_@C_g_:Fe_3_O_4_@C_g_-DTC

Using an ultrasonic for ten minutes, 0.5 g of Fe_3_O_4_@C_g_ was dispersed in a 30 mL solution of APTES in toluene (37 mmol L^−1^). The resultant solution was agitated at 40 °C for 48 h. Fe_3_O_4_@C_g_@NH_2_ was collected by separating the sediment from the solution using an external magnet, repeatedly washing it with toluene, and drying it in an oven at 60 °C. After 10 min of sonication, the sediment collected from the preceding stage was dispersed in 20 ml of methanol. After that, this solution was agitated for 12 h at room temperature with 2.2 mL of CS_2_. An external magnet was used to separate the final product of Fe_3_O_4_@C_g_-DTC from the mixture, and it was then washed with ethanol and allowed to dry overnight at 70 °C.

### Stabilization of Ag NPs over the surface of Fe_3_O_4_@C_g_-DTC

**Scheme 1:**
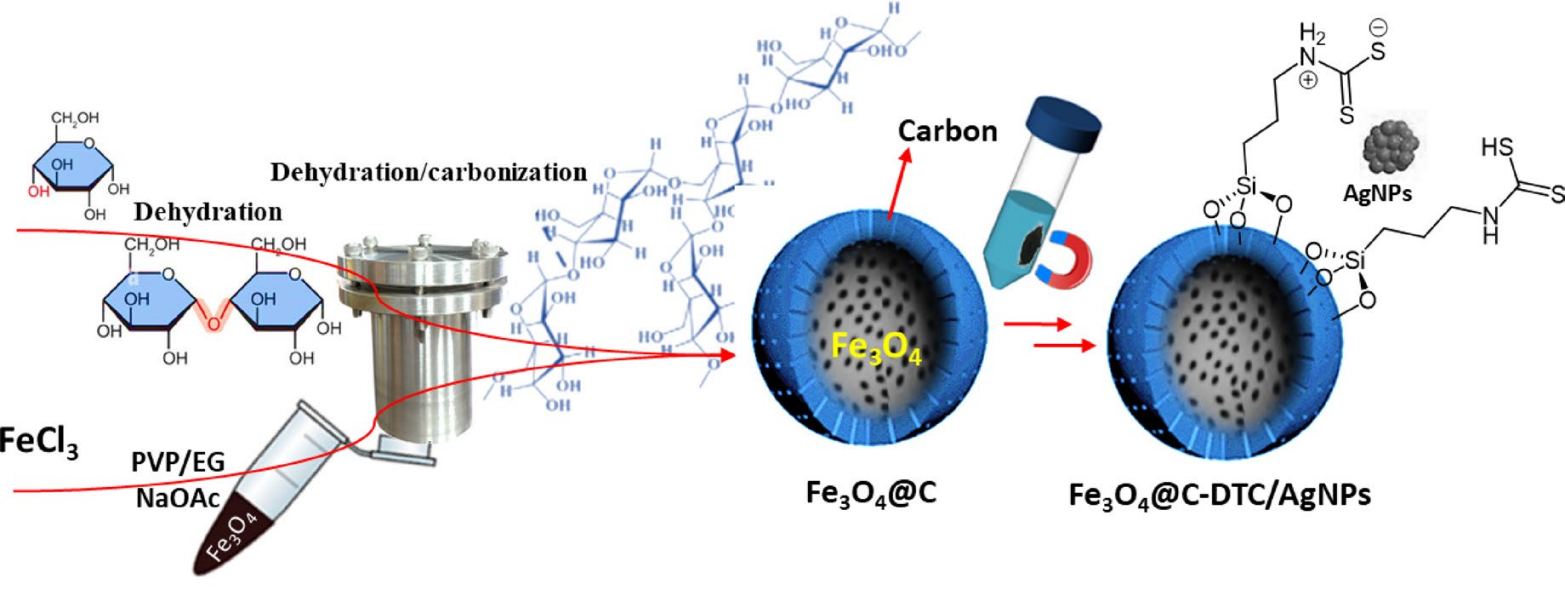
The experimental steps involved in the preparation and antibacterial applications of the Fe_3_O_4_@C_g_-DTC/AgNPs.

Initially, Fe_3_O_4_@C_g_-DTC (0.12 g) was ultrasonically dispersed for 15 min in 30 mL of deionized water. The resulting mixture was moved to the water bath and allowed to sit at room temperature for five minutes while mechanically stirring. After adding 100 mL of AgNO_3_ solution (0.82 mg mL^−1^), the solution was mixed for five mins at room temperature. 10 mL of NaBH_4_ solution was added dropwise for ten minutes under vigorous stirring and kept agitating for 5 h. At last, the Fe_3_O_4_@C_g_-DTC/AgNPs precipitate was collected and properly washed with deionized water, and the supernatant was disposed of. The final product was dried at 60 °C in the oven (Scheme 1).

### Investigation of antimicrobial effect

To investigate the antimicrobial effects of Fe_3_O_4_@C_g_-DTC/AgNPs composite, the macro broth dilution method was used to determine MIC (Minimum inhibitory concentration) and then MBC (minimum bactericidal concentration).

The studied bacteria included *E. coli* BAA 2340, *P. aeruginosa* BAA 3105, and *S. aureus* ATCC 43,300, cultured in nutrient broth and incubated for 24 h. Then, the microbial sediment was collected by centrifugation, and a microbial suspension equivalent to half of McFarland (1.5 × 10^8^ CFU/mL) was prepared.

In the next step, a suspension of this new composite was prepared in concentrations of 1, 2, 4, 8, 16, 32, 64, 128, 256, and 514 μg/mL in Muller Hinton Broth (MHB). Thus, 1 mL of MHB medium was added to 10 test tubes (labeled 1–10), and then 0.01 g of the prepared composite was dissolved in 1 mL of sterile distilled water. In this study, the first concentration of the studied composite was considered as 512 μg/mL for the antimicrobial assay. Subsequently, 204.8 μL of composite suspension was brought to a volume of 1 mL with MHB. Then 1 mL was added to the first tube, which contained 1 mL of medium. It means that the first tube had 2 mL of medium containing the desired composite. In the next step, 1 mL of the first tube was added to the second tube, and this was repeated in the same way until the last tube. In this way, serial dilutions were prepared. According to the initial results obtained, the starting concentration of the desired composite was different for the next series. The bacterial suspensions with turbidity equal to 0.5 McFarland (1.5 × 10^8^ CFU/mL) were diluted. Therefore, in each tube, a suspension of nanocomposite combined with bacterial suspension with final concentrations of 5 × 10^5^ CFU/mL was prepared. Subsequently, the tubes were incubated in a shaker incubation at 37℃ for 24 h at 160 rpm. Finally, the results were read, and MICs were determined. The positive control consisted of the bacterial suspension in MHB medium without the nanocomposite, to verify bacterial growth, while the negative control consisted of MHB medium with the nanocomposite but without bacteria, to ensure sterility of the assay. In the next step, to find the MBC of the desired nano-composite, 10 μL of the tube suspension with the lowest concentration that completely inhibited bacterial growth (no turbidity), along with the previous three tubes, was cultured on Mueller’s culture medium. After incubation (37 °C for 24 h), MBC values (99.99% of bacteria killed) were determined by counting the grown colonies.

### Investigation of biofilm inhibition properties

The biofilm inhibition properties were assessed following a previously described method, with slight modifications^32^. After determining the MIC and MBC, 100 µL of bacterial suspension and 100 µL of test concentrations below the MIC were added to a 96-well microtiter plate and incubated at 37 °C for 24 h. The wells were then emptied, dried, and fixed with 200 µL of diluted Fuchsin for 2 min. After staining, the wells were washed with PBS, and 200 µL of acetone-alcohol was added. After 5 min, the optical density was measured at 492 nm using an ELISA reader (Table S2).

### Cytotoxicity assessment

The reduction assay of the 3-(4,5-dimethyl-2-thiazolyl)-2,5-diphenyl-2*H*-tetrazolium bromide (MTT) was performed for the evaluation of cytotoxicity effects of Fe_3_O_4_@C_g_-DTC/AgNPs composite. Mouse embryonic fibroblast cells (NIH/3T3) were purchased and maintained in supplemented Dulbecco’s Modified Eagle Medium (DMEM) at 37 °C in 5% CO_2_ and 95% humidity. Briefly, NIH/3T3 cells were seeded in a 96-well plate at a concentration of 2 × 10^4^ cells/mL and incubated for 24 h. Then, different concentrations of composite (5, 15, 25, 50, 100, 200, 400 µg/mL) were added to each well. Doxorubicin (0.1 mM) and culture medium containing 1% DMSO were used as positive and negative controls, respectively. After 48 h of incubation, 20 µl of MTT solution (5 mg mL^−1^) were added to each well and incubated for an additional 3 h. Afterward, formazan crystals were dissolved in 150 µL DMSO, and the optical density (OD) values were measured by an ELISA reader at 570 nm. Finally, the OD of negative control wells (considered as a hundred percent) was used to calculate the cell viability percentage in test wells.

## Results and discussions

Here, Fe_3_O_4_@C_g_ was prepared as a cost-effective support for stabilizing Ag NPs. The Fe_3_O_4_ NPs with a mean diameter of 300 nm were prepared via a hydrothermal method at 200 °C. The as-prepared MNPs were added to an aqueous glucose solution and heated at 200 °C in an autoclave to prepare the carbonized MNPs. The carbonized MNPs were employed to support the stabilization of Ag NPs in the presence of NaBH_4_. Finally, various concentrations of the Ag NPs were stabilized over the surface of the Fe_3_O_4_@C_g_, and their antibacterial properties were studied (Scheme 1).

**Fig. 1:**
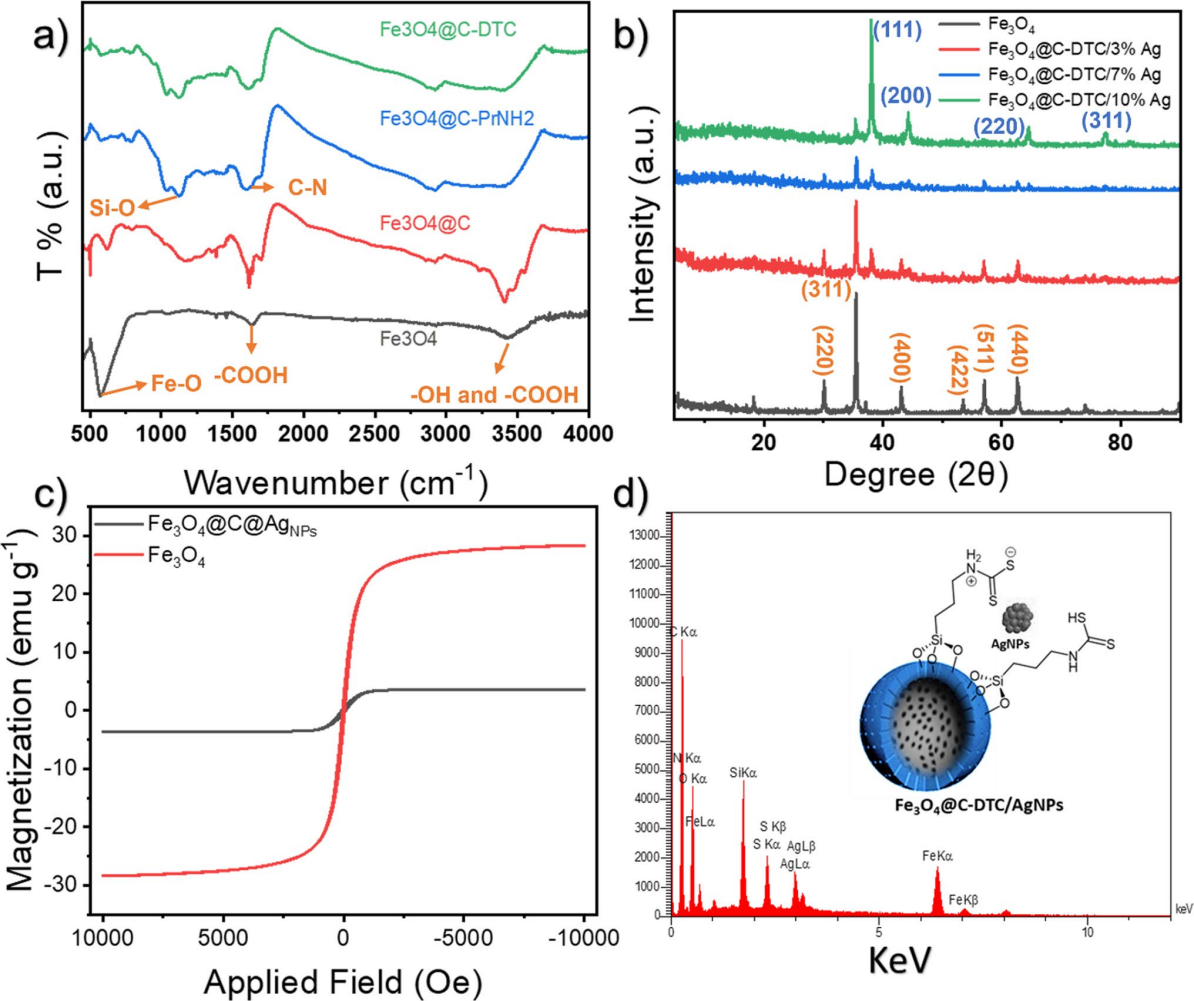
(**a**) The FT-IR spectra of the Fe_3_O_4_, Fe_3_O_4_@C_g_, Fe_3_O_4_@C_g_-NH_2_, and Fe_3_O_4_@C_g_-DTC. (**b**) The XRD pattern of the Fe_3_O_4_, Fe_3_O_4_@C_g_-DTC3% AgNPs (the blue numbers indicate the Miller indices of the Ag NPs and the orange numbers indicate the Miller indices of the Fe_3_O_4_ NPs), Fe_3_O_4_@C_g_-DTC7% Ag_NPs_, Fe_3_O_4_@C_g_-DTC10% Ag_NPs_. (**c**) The VSM analysis of the Fe_3_O_4_ and the Fe_3_O_4_@C_g_-DTC7% Ag_NPs_. (**d**) EDX analysis of Fe_3_O_4_@C_g_-DTC7% Ag_NPs_.

The FT-IR spectra of the bare and carbonized magnetic Fe_3_O_4_ NPs are shown in Fig. 1a. The FT-IR spectra of the bare Fe_3_O_4_ show a strong peak at 571 cm^−1^, attributed to the Fe–O bonds in the magnetic materials. The presence of the –OH groups over the surface of the magnetic material is evident from a wide peak at 3300–3600 cm^−1^. The FT-IR spectra of the Fe_3_O_4_@C_g_ show a drastic increase in the intensity of the –OH peak at 3300–3600 cm^−1^, indicating an increase in the –OH and –COOH content of the material. Also, sharp peaks at 1616 cm^−1^ suggest the presence of the acidic carbonyl groups in the final structure. A weak peak at 2923 cm^−1^ appeared in the FT-IR spectra of the final composite, indicating the presence of the aliphatic groups in the final composite. The FT-IR spectra of the amine functionalized material show a decrease in the intensity and a widening in the peak at 3000–3500 cm^−1^, indicating a decrease in the surface –OH groups due to the bonding with the Si of the APTES and an increase in the NH_2_ functional groups. The presence of the Si–O groups is evidenced by the appearance of new peaks at 1000–1100 cm^−1^. Additionally, the peaks at approximately 1600 cm^−1^ can be attributed to the presence of C–N bonding in APTES. The FTIR spectra of the dithiocarbamate functionalized composite are almost identical to the FT-IR spectra of the Fe_3_O_4_@C_g_@NH_2_ due to the overlay of the peaks of various functional groups and low concentration of dithiocarbamate groups. However, further characterizations were employed to characterize the presence of sulfur groups in the composite matrix.

The XRD patterns of the bare Fe_3_O_4_ and the Ag NPs decorated carbonized Fe_3_O_4_ are shown in Fig. 1b. The XRD pattern of the bare Fe_3_O_4_ shows characteristic peaks at 30.14, 35.47, 43.13, 53.5, 57.07, and 62.61 degrees corresponding to crystal planes of (220), (311), (400), (422), (511), and (440). This data shows a face-centered cubic (fcc) lattice structure for the Fe_3_O_4_ nanoparticles (JCPDS card no. 19-0629). The XRD patterns of the Fe_3_O_4_@C_g_-DTC3%Ag_NPs_, Fe_3_O_4_@C_g_-DTC7%Ag_NPs_, and Fe_3_O_4_@C_g_-DTC10%AgNPs are also represented in Fig. 1b. All the represented XRD patterns show the characteristic peaks of the Ag NPs at 2θ 38.06, 44.52, 64.52, and 77.4 degrees corresponding to crystal planes of (111), (200), (220), and (311), respectively, regarding the crystallographic planes of the face-centered cubic silver crystals. These XRD spectra also show the characteristics of the Fe_3_O_4_ NPs; however, an increase in the Ag content of the materials resulted in a sharp rise in the intensity of the Ag NPs and a decrease in the intensity of the Fe_3_O_4_ MNPs.

The VSM analysis of the bare and the functionalized Fe_3_O_4_ is conducted to study the magnetic properties of the synthesized materials. As shown in Fig. 1c, the magnetic content of the composite shows a drastic drop compared to the bare Fe_3_O_4_ due to the shielding effect of the carbon shell. The calculated saturation magnetization value (SMV) for the Fe_3_O_4_ and the Fe_3_O_4_@C_g_-DTC7%AgNPs was 28.36 and 3.59 emu g^−1^, respectively. Such a drastic drop in the SMV suggests that the functionalization process created a high carbon content over the surface of the Fe_3_O_4_.

The EDS analysis was performed to study the elemental composition of the final composite. As shown in Fig. 1d, the results of this study show the presence of Fe, O, C, Si, N, S, and Ag in the composite matrix. The EDS analysis shows a slightly lower concentration for the Ag NPs compared to the theoretical calculations, which is a normal phenomenon and a direct result of the washing step. The results of the EDS analysis show a 5.27, 4.85, 4.26, 41.20, 9.26, 7.62, 27.54 W% concentration for Ag, S, N, C, Si, Fe, O, respectively. These data prove the formation and the purity of the composite.

**Fig. 2:**
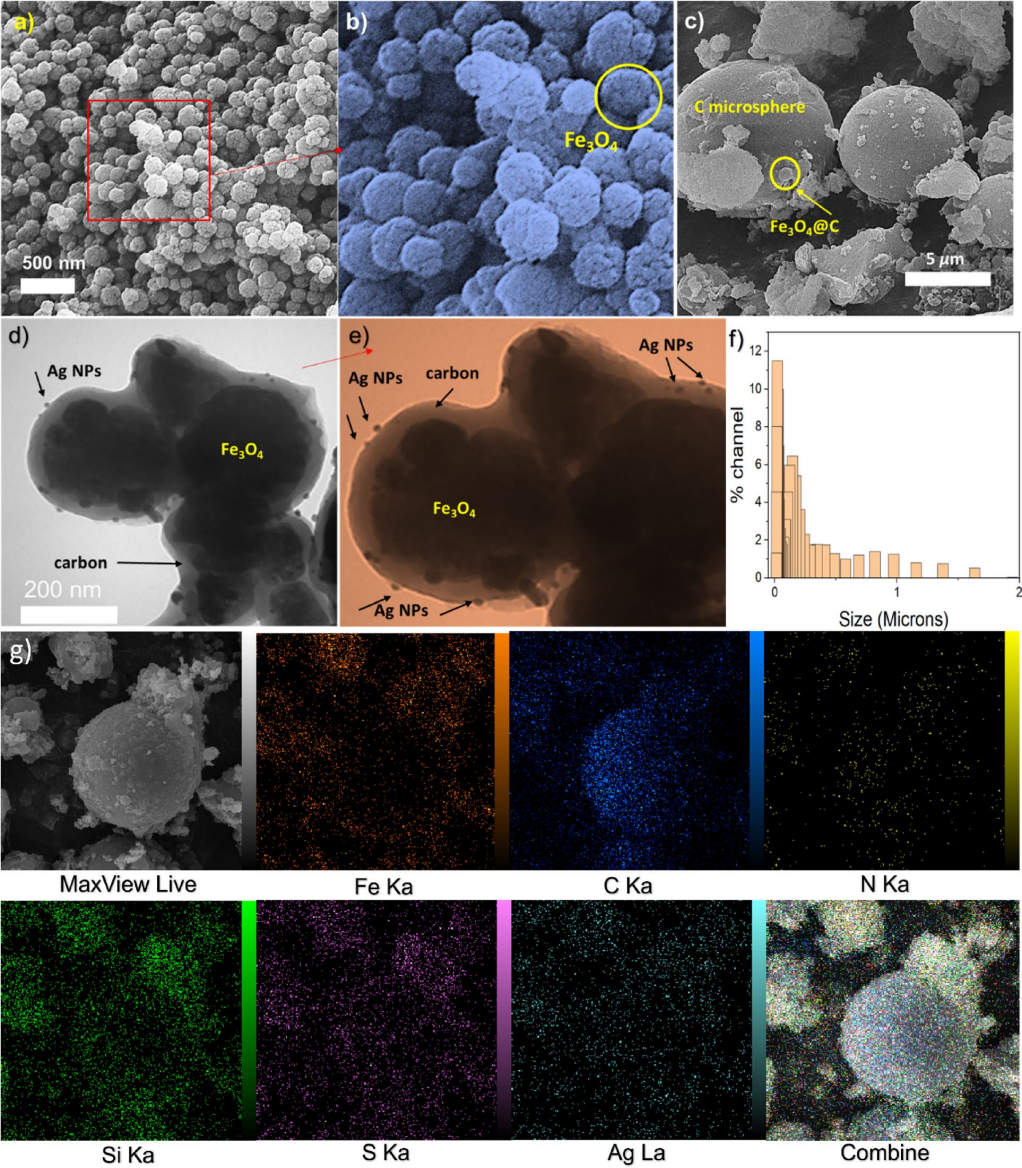
(**a**, **b**) The SEM image of the Fe_3_O_4_. (**c**) The SEM image of the Fe_3_O_4_@C_g_-DTC7% Ag_NPs_. (**d**, **e**) The TEM images of the Fe_3_O_4_@C_g_-DTC7% Ag_NPs_. (**f**) the size distribution of the Fe_3_O_4_@C_g_-DTC7% Ag_NPs_. (**g**) the mapping analysis of Fe_3_O_4_@C_g_-DTC7% Ag_NPs._

To study the morphology and the surface of the materials, the SEM and TEM techniques were employed (Fig. 2). The SEM image of the bare Fe_3_O_4_ NPs shows monodispersed material with spherical morphology and a mean diameter of approximately 300 nm (Fig. 2a, b). The SEM images of the Ag NPs decorated carbonized magnetic materials show that the Fe_3_O_4_@C_g_-DTC7%AgNPs adopted the spherical morphology of the bare Fe_3_O_4_. However, a drastic increase in the size of the NPs is evident due to the highly successful carbonization process. Moreover, the Fe_3_O_4_@C_g_-DTC7%AgNPs (Fig. 2c) lack the monodispersity of the parent Fe_3_O_4_. The TEM image of the Fe_3_O_4_@C_g_-DTC7%AgNPs shows a core–shell morphology for the magnetic composite in which the Fe_3_O_4_ acted as the core and the carbon acted as the shell (Fig. 2d, e). This image displays monodispersed Ag NPs with a size of less than approximately 10 nm distributed across the surface of the magnetic composite (Fig. 2f). Also, the average Ag nanoparticle size was determined by analyzing the TEM images using ImageJ software (N = 19). The analysis revealed a mean particle size of 9.83 ± 2.83 nm. This shows that the dithiocarbamate functionalities of the final composite perfectly helped the stabilization of the Ag NPs. To study the NPs size distribution, the DLS analysis was conducted, which further indicated that the carbonized MNPs lack monodispersity with a PDI of 11.81. The special elemental distribution and elemental composition of the proposed composite are studied by mapping analysis (Fig. 2g). This analysis indicates the purity of the composite and the presence of all the expected elements. Even distribution of the Ag elements throughout the composite further indicates the monodispersity of the nanoparticles over the surface of the magnetic composite. These data prove the formation of the catalyst as it was proposed.

Evaluating the antibacterial effects of Fe_3_O_4_@C_g_-DTC/AgNPs composite was one of the main interests of the present study. Results from the macro broth dilution test are tabulated in Table 1. The nanocomposite exhibited significant antibacterial effects against drug-resistant gram-positive (*S. aureus*) and gram-negative (*E. coli* and *P. aeruginosa*) bacteria. As seen in Table 1, the nanocomposite was more effective against *E. coli*. Similarly, in a study by Xia et al., the antibacterial effects of Fe_3_O_4_@C@/Ag were studied and approved (7)(6)(5)(4)^20^. They hypothesized that the antibacterial effect of the nanocomposites is enhanced by the carbon layer. The intermediate carbon increases and strengthens the absorption of bacteria to the surface of the nanocomposites, and Ag NPs act quickly and disrupt bacterial cells. Furthermore, Dehghan et al. reported a MIC of 6.25 mg mL^−1^ against *S. aureus* for Ag/Fe_3_O_4_ nanocomposites^33^. The antimicrobial activity of the nanocomposite designed in the present study was significantly higher than that reported in the mentioned study. This difference may also be related to factors such as the presence of the carbon layer, synthesis method, and materials used. The tolerance level MBC/MIC ratio is represented in Table S1 as well.

**Table 1:** MIC and MBC (µg mL^−1^) of Fe₃O₄@Cg-DTC/AgNPs against drug-resistant bacteria (All values in the table are reported as mean ± standard deviation (SD)).

Comp	*E. coli* (BAA 2340)		*P. aeruginosa* (BAA 3105)		*S. aureus* (ATCC 43300)	
	MICs	MBCs	MICs	MBCs	MICs	MBCs
Fe_3_O_4_@C_g_-DTC/AgNPs	0.58 ± 0.38	0.58 ± 0.38	1 ± 0	2 ± 1.73	4.67 ± 3.06	8 ± 0
Ciprofloxacin	32 ± 0	64 ± 0	16 ± 13.86	32 ± 0	16 ± 13.86	42.67 ± 18.48

The MIC and MBC concentrations of Fe_3_O_4_@Cg-DTC/AgNPs in all bacteria were lower than those of ciprofloxacin. Based on the results, the antibacterial effect of the nanocomposite is remarkably better than that of ciprofloxacin. Ciprofloxacin, a fluoroquinolone antibiotic, is commonly used for the prevention of post-PB infections. Unfortunately, susceptibility to ciprofloxacin is diminishing^34^. Therefore, the development of new antibiotics or drugs including nanomaterials seems to be crucial. Applying nanoscale materials can result in more contact between the microbes and compounds. They also improve the absorption, bioavailability, and release of the drug into the bacteria^35^.

Ag NPs can interfere with bacterial cell membranes, impairing metabolism, respiration, and proliferation^35^. However, silver accumulation in the body can be harmful^36^. Therefore, different nanocomposites of Ag NPs with improved antibacterial properties and reduced cytotoxicity should be designed. Monica et al. reported that bio-nanocomposites of chitosan-coated Ag NPs are more effective than uncoated Ag NPs against *S. aureus*^37^. The stabilized Ag NPs in such composites prevent nanoparticle aggregation. The formation of larger particles greatly reduces the activity of silver nanoparticles^38^.

Sterilizing is vital in the PBs. Gillespie et al. reported an outbreak of post-PB infections caused by a needle contaminated with *P. aeruginosa*. Proper cleaning and sterilization of PB equipment are essential^39^, but biofilm formation poses challenges. Biofilms are aggregations of microbes that can adhere to the surfaces of medical devices^40‚41^. Owing to the low susceptibility of biofilms to antibiotics and disinfectants, new strategies must be employed to combat biofilms^43,42^. In the present study, the biofilm inhibition activity of nanocomposites was also evaluated. According to the results, the lowest concentration at which the Fe_3_O_4_@C_g_-DTC/AgNPs can inhibit biofilm formation in *E. coli* was equal to 0.0625 µg mL^−1^. Meanwhile, the concentrations required for *P. aeruginosa* and *S. aureus* were equal to 0.5 and 1 μg mL^−1^, respectively. Anti-biofilm activity of Ag NPs has been studied and demonstrated in various studies^46,44,45‚47^. Kalishwaralal et al. reported that 95% of biofilm formation of *P. aeruginosa* and *Staphylococcus epidermidis* was impeded by Ag NPs.

**Fig. 3:**
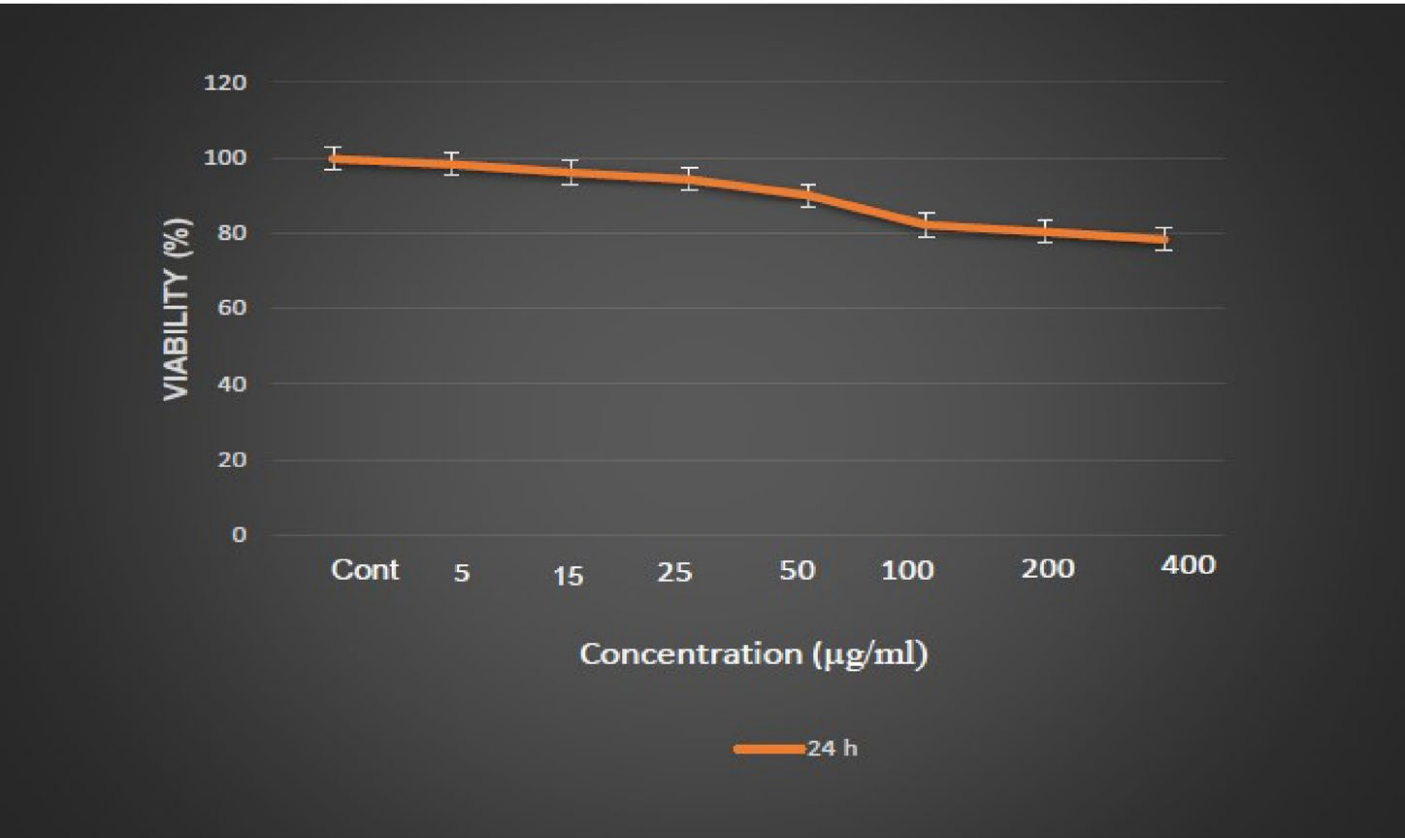
Percentage of cell viability in different concentrations of Fe_3_O_4_@C_g_-DTC/AgNPs.

While Ag NPs have potent antibacterial effects, they can cause lipid peroxidation, DNA damage, and cell apoptosis . Hence, there is concern about the use of Ag NPs. In one study, cell viability was reported to be almost twenty percent at 10 µg mL^−1^ of Ag NPs, but no reduction was observed at lower concentrations^49^. Also, in another study by Salomoni et al., the viability was reduced by 5.0 µg mL^−1^. Hence, evaluating the cytotoxicity effects of new Ag NP-based nanocomposites seems indispensable. In this study, the viability of cells in the presence of Fe_3_O_4_@C_g_-DTC/AgNPs was evaluated by MTT assay and is rendered in Fig. 3. Different concentrations of Fe_3_O_4_@C_g_/PrNHCS_2_AgNPs led to dose-dependent inhibition of the growth of NIH-3T3 cells. As illustrated in Fig. 3, the new nanocomposite did not induce cytotoxicity in NIH-3T3 cells, although a twenty-two percent decrease in cell viability was observed at 400 µg mL^−1^. Also, the cytotoxicity of Fe_3_O_4_@C_g_-DTC/AgNPs against the Vero cell line (normal cells) was evaluated in a study. In contrast to our research, a significant decrease in cell viability was observed in concentrations higher than 100 µg mL^−1^.

## Conclusion

Here, we created a magnetic composite functionalized with core–shell dithiocarbonate carbon and used it as a basis for Ag NP stabilization. The characterization demonstrates that the MNPs functionalization created an appropriate surface for Ag NP stabilization. The functionalization caused a thick coating of carbon to grow on the surface of the magnetic nanoparticles, which decreased the Fe_3_O_4_ monodispersity and magnetic content. Next, we evaluated the antibacterial and biofilm inhibition effects of this nanocomposite. The minimum inhibitory concentrations (MICs) against *Escherichia coli*, *Pseudomonas aeruginosa*, and *Staphylococcus aureus* were 0.58 ± 0.38, 1, and 4.67 ± 3.05 µg mL^−1^, respectively. The results demonstrated that Fe_3_O_4_@Cg-DTC/AgNPs exhibited stronger antibacterial activity than ciprofloxacin, a commonly used fluoroquinolone antibiotic. Furthermore, the nanocomposite displayed a high biofilm inhibition capability, which is crucial in preventing bacterial infections. Cytotoxicity assessments confirmed that the nanocomposite was biocompatible with NIH-3T3 cell lines, highlighting its potential for therapeutic applications. Given the rising threat of antibiotic resistance, this nanocomposite could serve as a promising alternative to conventional antibiotics.

## Supplementary Information

Below is the link to the electronic supplementary material.

Supplementary Material 1

## Data Availability

All data generated or analyzed during this study are included in this published article.
