# Supplementary material for: Layer by layer preparation of Fe3O4@Cg-DTC/AgNPs as colloidal antimicrobial and anti-biofilm agent
Source: Sci Rep. 2025 Dec 2;16:519. doi: 10.1038/s41598-025-29960-w (PMC12775416; doi:10.1038/s41598-025-29960-w)
Supplement: Supplementary file 1 — Supplementary Material 1 [file 41598_2025_29960_MOESM1_ESM.pdf]

Supporting Information

Table S1. Indicate the tolerance level MBC/MIC ratio in Table 1 as well. Typically, an MBC/MIC ratio that signifies the $Fe3O4$@Cg-DTC/AgNPs is inhibitory or bactericidal.

Bacteria	MIC (μg/mL)	MBC (μg/mL)	MBC/MIC Ratio	Tolerance Level / Classification
S. aureus (ATCC 43300)	4.67 ± 3.05	8	≈1.71	Bactericidal (Strong) - Ratio ≤ 2; Indicates strong killing effect, not just inhibitory.
P. aeruginosa (BAA 3105)	1	2 ± 1.73	=2	Bactericidal (Strong) - Ratio = 2; Indicates strong killing effect, not just inhibitory.
E. coli (BAA 2340)	0.58 ± 0.38	0.58 ± 0.38	=1	Bactericidal (Strong) - Ratio = 1; Indicates strong killing effect, not just inhibitory.

Table S2. The effective concentrations for biofilm inhibition

E. coli		*P. aeruginosa*		*S. aureus*	
Concentration	Biofilm (%)	Concentration	Biofilm (%)	Concentration	Biofilm (%)
0.25	0 ± 0	0.5	0.00 ± 0.00	2	0.00 ± 0.00
0.125	0 ± 0	0.25	46.00 ±1.00	1	0.00±0.00
0.0625	0 ± 0	0.125	54.33±2.08	0.5	47.67±3.51
0.03125	53.67 ± 1.53	0.0625	98.33 ±1.53	0.25	78.67±2.08
